# Responses of Macroinvertebrate Communities to Heterogeneity Among Typical Reaches in the Upper Yangtze River

**DOI:** 10.1002/ece3.72439

**Published:** 2025-11-10

**Authors:** Yang Wei, Wang Si‐Yuan, Zhang Xian‐Bing, Zhang Yang‐Chun, Chen Zi‐Wei, Yang Zhong‐Chao, Yang Sheng‐Fa, Chen Qi‐Liang, Xie Jia‐Hui, Tan Li‐Wei

**Affiliations:** ^1^ Chongqing Jiaotong University Chongqing China; ^2^ National Engineering Research Center for Inland Waterway Regulation Chongqing China; ^3^ Yangtze Three Gorges Technology & Economy Development Co., Ltd Beijing China; ^4^ Chongqing Normal University Chongqing China

**Keywords:** environmental heterogeneity, flow velocity, macroinvertebrates, sediment nutrients, Yangtze River

## Abstract

Benthic macroinvertebrates are important indicators of river ecosystem health, and their spatial distribution is highly sensitive to environmental heterogeneity. In large mountainous rivers characterized by complex geomorphology and strong hydrodynamics, habitat structural diversity may influence community structure through multi‐pathway mechanisms. This study focused on three representative reaches of the upper Yangtze River—the Canyon, Anabranching, and Reef–Tuo reaches. By integrating two‐dimensional hydrodynamic simulations, in situ measurements of water and sediment conditions, and benthic community surveys, we systematically analyzed the effects of environmental factors on community composition. Non‐metric multidimensional scaling (NMDS) and redundancy analysis (RDA) revealed significant differences in community richness, diversity, and functional group composition among reaches. Partial least squares path modeling (PLS‐PM) further demonstrated a “dual‐pathway” ecological mechanism: flow velocity not only directly promoted the abundance of rheophilic taxa but also indirectly influenced the distribution of sediment‐preferring taxa by altering sediment condition. Hydrodynamic simulation results further validated the spatial basis of this mechanism. Flow velocity gradients were strongly shaped by geomorphic features, and notably, stable low‐velocity zones persisted in the Anabranching and Reef–Tuo reaches. These areas served as important refugia for benthic communities, reinforcing the ecological applicability of the “dual‐pathway” mechanism in complex river segments.

## Graphical abstract

This study examines how hydraulic and geomorphological heterogeneity shape benthic macroinvertebrate communities in the mountainous upper Yangtze River. Integrating field measurements with hydrodynamic modeling, it identifies distinct community patterns across canyon, anabranching, and Reef–Tuo reaches, driven by flow velocity, sediment dynamics, and nutrient retention. Findings underscore both direct and indirect pathways through which physical habitat structure influences biodiversity.

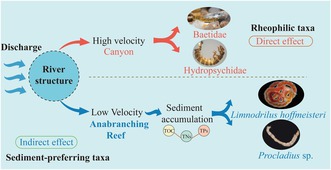

## 1 Introduction

Large river ecosystems are among the most biodiversity‐rich systems on Earth, playing an irreplaceable role in nutrient cycling, sediment transport, and habitat provision. However, these structurally complex and functionally diverse systems are increasingly threatened by both natural and anthropogenic stressors, placing their ecological integrity and systemic stability at risk. Among aquatic taxa, benthic macroinvertebrates are widely regarded as key bioindicators due to their sensitivity to environmental change (Zhao et al. 2024), functional roles in aquatic food webs, and strong habitat dependence (Li et al. 2020; Tampo et al. 2021).

Benthic macroinvertebrates are closely linked to the structure and dynamics of their surrounding habitats, making their distribution highly sensitive to environmental heterogeneity (Romoth et al. 2023). In small and medium‐sized streams, researchers have commonly adopted microhabitat‐based classification schemes—such as riffle–pool units—to explore spatial variations in community composition (Wang et al. 2023). However, such fine‐scale typologies often fall short in capturing the complexity and variability of hydrodynamic and geomorphic patterns in large mountainous rivers, where structural heterogeneity operates across broader spatial scales. This mismatch limits the ability to generalize ecological findings from localized habitats to broader management contexts. Accordingly, there is an urgent need to develop reach‐scale classification frameworks that better reflect the dominant geomorphic features and hydrological gradients of large river systems, thereby facilitating a more representative understanding of community–environment relationships.

In recent years, numerous studies have demonstrated that factors such as flow velocity and substrate composition exert significant control over benthic community structure in microhabitats or small to medium‐sized streams (Theodoropoulos et al. 2017; Vadher et al. 2017; Kędzior et al. 2022). Building on this foundation, some studies have attempted to scale up ecological investigations to broader spatial extents, yet most remain limited to observations of river reaches of a single type, making it difficult to fully capture community responses under natural high‐energy environments (Sotomayor et al. 2025). In contrast, large mountainous rivers exhibit pronounced geomorphic complexity and dynamic hydrodynamic processes, with distinct reach types—such as confined canyons, anabranching channels, and Reef–Tuo zones—showing substantial differences in hydraulic gradients, substrate composition, and spatial connectivity (Yang et al. 2023). This reach‐scale environmental heterogeneity may profoundly influence the functional composition and spatial organization of benthic communities by altering habitat configurations and ecological niche distributions (Wang et al. 2023; Richards et al. 2018; Bouska et al. 2023). However, systematic empirical studies on how these distinct reach types collectively structure benthic communities remain extremely limited—particularly in large mountainous rivers across Asia. Most current studies still rely on static descriptions or single‐factor correlations, lacking integrative, mechanism‐based frameworks that combine numerical simulations (e.g., flow field modeling) with ecological field surveys to reveal causal linkages between physical processes and biological responses (Becquet et al. 2022). There is an urgent need to establish more representative ecological research frameworks at the reach scale to deepen our understanding of biodiversity maintenance mechanisms under complex hydro‐geomorphic conditions.

In response to the aforementioned research needs, this study conducted a systematic field investigation in a geomorphologically diverse segment of the upper Yangtze River within the Three Gorges Reservoir region, aiming to explore how habitat heterogeneity shapes benthic macroinvertebrate community structure. Three representative reach types commonly found in large mountainous river systems—Canyon reaches, Reef–Tuo reaches, and Anabranching reaches—were selected as focal units. Field sampling was conducted under hydrologically representative conditions (near the multi‐year mean discharge), accompanied by quantitative measurements of key environmental factors to characterize spatial variability in flow velocity, turbidity, and substrate composition across the different reaches. These environmental variables were then analyzed in relation to benthic community structure to assess the potential contribution of habitat heterogeneity to ecological resilience under hydrological disturbances. To further interpret community patterns from a hydrodynamic perspective, two‐dimensional flow simulations were conducted under multiple discharge scenarios, compensating for the limitations of single‐condition field sampling. By integrating ecological observations with hydrodynamic modeling, this study seeks to advance the understanding of biodiversity maintenance mechanisms and habitat‐ecological function relationships in large, high‐energy mountainous river systems.

## 2 Materials and Methods

### 2.1 Study Area and Sampling Design

The field survey of benthic macroinvertebrates was conducted along the upper Yangtze River between Fuling and Fengdu (29.72°–29.89° N, 107.37°–107.75° E) during June 2024. Prior to this study, we conducted preliminary surveys in this river reach during 2020–2023, which provided the methodological and data foundation for the present systematic investigation. Based on this foundation, the present study selected a hydrologically representative period for mountainous rivers to systematically expand the number and spatial distribution of sampling sites. A reach‐scale analysis was then conducted to examine how benthic community structure responds to habitat heterogeneity across different geomorphic settings. This period was characterized by stable flow conditions and water temperatures ranging from 22°C to 30°C, which fall within the optimal range for the growth and reproduction of benthic macroinvertebrates. This pre‐flood sampling window was scientifically established as optimal for capturing baseline community structures in fluvial ecosystems (Li et al. 2025).

The investigated river section represents a large mountainous trunk river system characterized by alternating wide‐valley and canyon sections, with channel widths ranging from 400 to 1600 m and water depths exceeding 110 m in some areas. This geomorphologically dynamic system exhibits pronounced fluvial heterogeneity in geomorphology in bed structure, particularly in substrate composition and hydraulic regimes (Zhu et al. 2023). Based on comprehensive hydrogeomorphic criteria, three distinct reach types were identified: (1) Canyon reach, confined by bedrock walls, uniform flow velocities, and cobble‐dominated substrates; (2) Anabranching reach, characterized by multi‐thread channels partitioned by mid‐channel gravel bars, displaying reduced velocities and heterogeneous sand‐gravel substrates; (3) Reef–Tuo reach, formed downstream of submerged bedrock outcrops, featuring channel widening with pool and fine sediment accumulations.

Nine spatially independent sampling transects were selected within each of three dominant reach types (Canyon, Anabranching, Reef–Tuo) within the investigated area to systematically assess the reach‐scale community structure of benthic macroinvertebrates and their habitat characteristics. To mitigate depth‐related distortions during inter‐habitat analyses, each sampling transect featured six vertically stratified collection sites positioned along an orthogonal transect extending from riparian zones to the thalweg, aligned with predetermined bathymetric parameters (0.5, 2.0, 4.0, 8.0, 15.0, and 30.0‐m intervals). Benthic organisms were quantitatively sampled at all sites using a Peterson grab sampler (0.0625 m^2^ effective sampling area) following established protocols (Yi et al. 2018). Sampled materials were immediately transferred to pre‐chilled containers (4°C) and transported to the laboratory within 12 h for taxonomic identification and biomass quantification.

Concurrent with benthic organism collection, surficial substrate samples were systematically acquired for nutrient characterization. At each sampling transect, duplicate sediment cores (0–10 cm depth) were extracted adjacent to biological sampling locations using a modified Peterson grab sampler.

Water quality assessments were conducted synchronously with benthic sampling across all transects. Three sampling sites positioned 5.0, 10.0, and 30.0 m from the riverbank along each cross‐section were continuously monitored at 0.5 m below the water surface. A calibrated multiparameter probe (Hydrolab DS5X, Hach Inc., USA) recorded temperature (°C), dissolved oxygen (mg/L), pH, specific conductivity (*μ*S/cm), chlorophyll‐a (*μ*g/L), and turbidity (NTU) with ±2% measurement accuracy. Composite water samples (1 L) were collected at each sampling site using a Van Dorn sampler, preserved at 4°C in amber glass bottles, and transported to the laboratory within 12 h for dissolved nutrient analysis.

Concurrent velocity measurements employed a 200 kHz Acoustic Doppler Current Profiler (ADCP; RDI 40, Teledyne RD Instruments) deployed at three hydrodynamic monitoring points per transect (Das and Debnath 2025).

### 2.2 Laboratory Analyses of Macroinvertebrate and Sediment Samples

#### 2.2.1 Laboratory Analyses of Macroinvertebrates

The collected sediment samples were initially processed through a 500 *μ*m mesh sieve to eliminate non‐biological particles (Wang, Bao, et al. 2024; Wang, Guo, et al. 2024), followed by fixation in 10% formalin solution to preserve morphological integrity (Tamura and Kagaya 2016). Taxonomic identification was conducted under a stereomicroscope (Olympus SZX16), with classification referencing internationally recognized taxonomic systems (e.g., ITIS) and authoritative Chinese literature, including *Fauna Sinica* and *Atlas of Aquatic Organisms in Chinese Basins* (Li et al. 2007; Wang 2020; Zhang and Li 2019). More than half of the taxa were identified to genus or species level, with the remainder classified to higher taxonomic ranks (family) when morphological characteristics were insufficient for finer resolution. The taxonomic resolution for each recorded taxon is indicated in Table 1, where genus and species names are italicized and “sp.” denotes an unidentified species within a known genus.

**TABLE 1:** List of macroinvertebrates and their distribution in the three typical river sections.

Number	Species	Canyon reach			Reef–Tuo reach			Anabranching reach			Habit
		C1	C2	C3	T1	T2	T3	A1	A2	A3	
Oligochaeta											
Tubificida											
A	*Limnodrilus hoffmeisteri*	−	+	−	−	+	−	+*	+*	−	ST
B	*Limnodrilus grandisetosus*	−	−	−	+	−	−	−	+*	+*	ST
C	*Branchiura sowerbyi*	−	−	−	−	+*	+*	−	−	+	ST
D	*Tubifex tubifex*	−	−	−	−	−	−	++*	+*	−	ST
E	Naididae	−	−	−	+*	+*	+*	++*	++*	+*	ST
Insecta											
Diptera											
F	*Polypedilum* sp.	−	−	−	+*	+*	−	+*	+*	−	ST
G	*Orthocladius* sp.	−	−	−	−	−	−	+*	−	+*	RT
H	*Procladius* sp.	−	+	−	−	++*	++*	+*	−	+*	ST
I	*Chironomus* sp.	−	−	−	−	−	−	+*	+*	−	ST
J	*Cryptochironomus* sp.	−	−	−	−	−	+	−	+*	+*	ST
Trichoptera											
K	Rhyacophilidae	−	−	−	−	+*	+*	+*	+*	++*	RT
L	Hydropsychidae	+*	−	+*	−	−	−	−	+*	+*	RT
M	Psychomyiidae	−	−	−	−	−	−	−	+*	+*	ST
N	Brachycentridae	−	−	−	+*	++*	+*	−	+*	+*	RT
Ephemeroptera											
O	Baetidae	++*	++*	++*	+*	+*	−	+*	+*	+*	RT
P	Heptageniidae	−	−	−	−	−	−	+*	−	+*	RT
Q	Siphlonuridae	−	−	−	+	−	−	−	−		RT
Plecoptera											
R	Capniidae	+	−	−	−	−	−	+*	+*	+*	RT
S	Perlodidae	−	−	−	−	−	+	+*	−	+*	RT
Malacostraca											
Amphipoda											
T	*Gammarus sinensis*	+*	+*	+*	++*	++*	++*	++*	++*	+*	RT

*Note:* “sp.” unidentified species within a known genus; “−” Not found; “+” Average density < 40 ind/m^2^; “++” Average density ≥ 40 ind/m^2^; “*” Dominant species; “ST” Sediment‐preferring taxa; “RT” Rheophilic taxa.

Individual counts for each taxon were recorded and standardized to density values (ind./m^2^) using the sampler's effective area (0.0625 m^2^). To comprehensively characterize habitat‐specific community patterns, depth‐stratified samples within individual transects were pooled, generating composite datasets for subsequent multivariate analyses.

#### 2.2.2 Laboratory Analyses of Sediment Samples

Water quality analysis parameters, including permanganate index (COD_Mn_), total nitrogen (TN), total phosphorus (TP), ammonium nitrogen (NH₄^+^‐N), and suspended solids (SS), were quantified (The State Environmental Protection Administration 2002). For sediment nutrient characterization, air‐dried samples were homogenized and sieved through a 60‐mesh (250 μm) stainless steel sieve to remove plant residues and coarse debris. Total organic carbon (TOC) was analyzed via potassium dichromate oxidation titration, while total nitrogen (TNs) and total phosphorus (TPs) in sediments were determined using the semi‐micro Kjeldahl method and molybdenum–antimony anti‐spectrophotometric method, respectively, as prescribed in the aforementioned national standard.

### 2.3 Hydrodynamic Numerical Modeling

**FIGURE 1:**
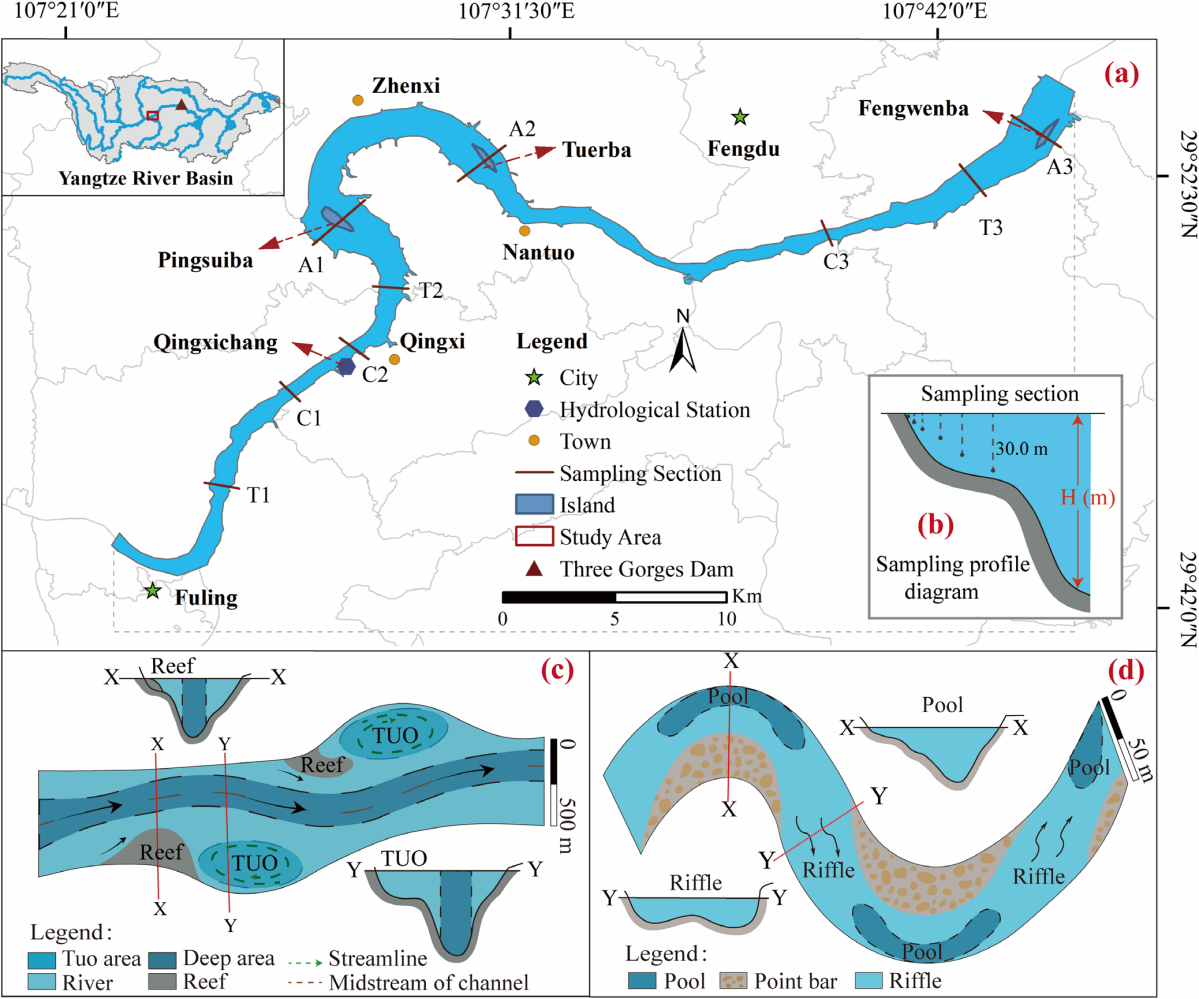
Study area and sampling layout in the upper Yangtze River. Nine benthic sampling sections (A1–A3, T1–T3, and C1–C3) were selected to represent three typical habitat types: Anabranching reaches, Reef–Tuo reaches, and Canyon reaches. (a) Map showing the location of the study area within the Yangtze River Basin. (b) Schematic diagram of the sampling section, where benthic organisms were collected at six water depths (0.5 m, 2 m, 4 m, 8 m, 15 m, and 30 m) along the vertical water column at each sampling section. (c) depicts the Reef–Tuo habitat defined in this study, consisting of submerged reef structures and adjacent low‐velocity Tuo zones that collectively form a distinct deep‐water environment. In comparison to the conventional riffle–pool morphology, the Reef–Tuo habitat exhibits greater geomorphological complexity and generates a spatially heterogeneous deep‐water system. (d) illustrates a typical riffle–pool habitat, characterized by the sequential alternation of shallow riffles and deeper pools along the riverbed.

To characterize benthic habitat hydraulics (flow velocity, water depth, flow regime), this study established a high‐resolution digital elevation model (DEM) of the Fuling–Fengdu river reach (Yangtze River, Figure 1) using 1:500 bathymetric survey data from the Chongqing Waterway Engineering Survey and Design Institute (June 2015). The DEM explicitly preserved natural microtopographic features, including thalweg channels, shallow water near the river bank, and mid‐channel islands, providing critical geomorphic inputs for hydrodynamic simulations.

The MIKE 21 hydrodynamic module (DHI, Denmark) was implemented with unstructured triangular meshes (mean edge length = 20 m; 5 m refinement in critical zones) and 10 sigma‐layered vertical discretization. Model parameters were calibrated using June 2020 field‐measured water levels and velocity profiles, achieving satisfactory agreement (RMSE ≤ 5%) between simulated and observed values. Three characteristic discharge scenarios were analyzed: Low‐flow regime (*Q*_min_ = 6800 m^3^/s), Mean‐flow regime (*Q*_mean_ = 12,500 m^3^/s), High‐flow regime (*Q*_max_ = 21,300 m^3^/s).

Hydrological boundary conditions were derived from Qingxichang gauging station's multiyear June records. As field sampling occurred under near‐mean discharge conditions (*Q* = 12,300 m^3^/s), subsequent ecohydraulic analyses primarily utilized *Q*_mean_ simulation outputs to establish benthic community–environment relationships.

### 2.4 Data Processing and Analysis

#### 2.4.1 Analysis of Biodiversity Indices

To assess and compare benthic macroinvertebrate diversity across reach‐scale habitat types, five complementary indices were employed: dominance index (*Y*; Equation 1), Margalef richness index (*D*_M_; Equation 2), Shannon–Wiener diversity index (*H*′; Equation 3), Simpson diversity index (*D*; Equation 4), and Pielou evenness index (*J*; Equation 5) (Guan et al. 2024) Previous studies have shown that diversity metrics calculated at higher taxonomic levels (e.g., genus or family) are generally comparable to species‐level analyses when evaluating overall community patterns and environmental gradients (Pires et al. 2021; de Oliveira et al. 2020). Therefore, in this study, each taxon was treated as an operational taxonomic unit and diversity indices were calculated using the lowest taxonomic resolution available for each taxon (species, genus, or family). This approach allows taxa identified at different taxonomic levels to be integrated into the analysis without loss of ecological information, ensuring that diversity indices reliably reflect community composition patterns across habitat types. This method is consistent with approaches used in other ecological studies (Min and Kong 2020; Liu et al. 2021).

(1) $$Y=\left(n_{i}/N\right)\times f_{i}$$

(2) $$D_{M}=\left(S-1\right)/\ln N$$

(3) $$H^{\prime }=-\sum_{i=1}^{S}P_{i}\ln P_{i}$$

(4) $$D=1-\sum_{i=1}^{S}{P_{i}}^{2}$$

(5) $$J=H^{\prime }/\ln S$$

The symbols in the above equations are defined as follows: *nᵢ* is the number of individuals of species *i*; *N* is the total number of individuals across all species; *S* is the total number of species recorded in the sampling area; *f*_i_ represents the occurrence frequency of the species at various points; and *pᵢ* is the relative abundance of species *i*, calculated as *pᵢ = nᵢ*/*N* and *Y* > 0.02 is defined as the dominant species.

Diversity metrics were computed in Canoco 5. Differences among habitat types (Canyon reach, Anabranching reach, and Reef–Tuo reach) were assessed using one‐way ANOVA in SPSS 26.0. Data were tested for normality (Shapiro–Wilk test) and homogeneity of variance (Levene's test) before analysis. Tukey's HSD was used for post hoc comparisons. A significance level of *p* < 0.05 was adopted for all statistical tests (Munyai et al. 2024).

#### 2.4.2 Community Structure Analysis

To explore spatial variation in benthic macroinvertebrate communities across different habitat types, non‐metric multidimensional scaling (NMDS) was applied to visualize the community structure (Milner et al. 2022). NMDS was used to visualize community structure differences, with stress < 0.2 indicating satisfactory ordination quality (Guan et al. 2024). Using Bray–Curtis dissimilarity matrices computed in the “*vegan*” package (R), ANOSIM (Esmaeili Ofogh et al. 2023) and PERMANOVA (Sofi et al. 2022) quantified significant compositional differences among reach types (both *p* < 0.001; 999 permutations), providing robust statistical support for the spatial differentiation of benthic communities.

#### 2.4.3 Statistical Analyses

For environmental variables, one‐way ANOVA was applied when the assumptions of normality and homogeneity of variance were satisfied; otherwise, the Kruskal–Wallis test was used (Mercier et al. 2022). The Kruskal–Wallis test, as a rank‐based non‐parametric method, is distribution‐free and robust to skewness and outliers, thereby providing reliable inference when parametric assumptions are not met. The complementary application of these two tests ensured methodological consistency and statistical robustness in group comparisons across variables with heterogeneous distributional properties.

#### 2.4.4 Association Analysis of Community Structure With Environmental Variables

First, we used redundancy analysis (RDA) (Sun et al. 2024) to explore how benthic macroinvertebrate communities respond to environmental gradients. RDA was selected as an appropriate constrained ordination technique for directly quantifying the relationships between community composition and measured environmental variables, thereby enabling identification of the primary environmental drivers and visualization of their effects on community structure. Prior to analysis, abundance data were Hellinger‐transformed to reduce the influence of zero inflation and enhance linearity (Li et al. 2021). Environmental variables were log(*x* + 1)‐transformed and standardized to improve comparability (Muluye et al. 2023). Significant predictors (*p* < 0.05) were selected using Monte Carlo permutation tests (999 permutations) and retained in the final model.

Finally, to clarify the direct and indirect pathways through which spatial and environmental factors affect benthic organisms, we used the “*plspm”* R package to construct a Partial Least Squares Path Modeling (PLS‐PM) structural equation model and plotted the pathways (Xie et al. 2024; Nie et al. 2025). This model allows for the simultaneous use of observed variables (outer model) and latent variables (inner model) to evaluate the fit of the predictive model in a complex ecological system. The PLS‐PM model not only reveals causal relationships between observed variables (including positive, negative, or no correlation), but also characterizes the contribution of latent variables to observed variables and the overall model fit, thus helping to optimize the model and identify the best linear predictive relationship. All analyses were performed in R version 4.3.0.

## 3 Results

### 3.1 Community Composition Characteristics of Benthic Macroinvertebrates

A total of 20 species of benthic macroinvertebrates were identified from the nine sampling sites, encompassing 6 taxonomic orders: Tubificida, Diptera, Trichoptera, Ephemeroptera, Plecoptera, and Amphipoda (Table 1). It is worth noting that no Mollusks were recorded in the quantitative samples. This absence reflects the in‐channel soft‐substrate sampling method, which did not cover epilithic or littoral habitats where Mollusks typically occur. However, Mollusks were qualitatively observed on shoreline rocks during fieldwork (see Figure 1); as these locations fell outside the defined sampling scope, they were not included in the present analysis.

The overall mean density of benthic macroinvertebrates in the study area was 206.22 ± 102.90 ind/m^2^. Clear differences were observed among the three habitat types. The Anabranching reach (AR) exhibited the highest density (325.33 ± 18.47 ind/m^2^), followed by the Reef–Tuo reach (TR) (202.66 ± 24.44 ind/m^2^), and the lowest was recorded in the Canyon reach (CR) (90.66 ± 9.23 ind/m^2^).

**FIGURE 2:**
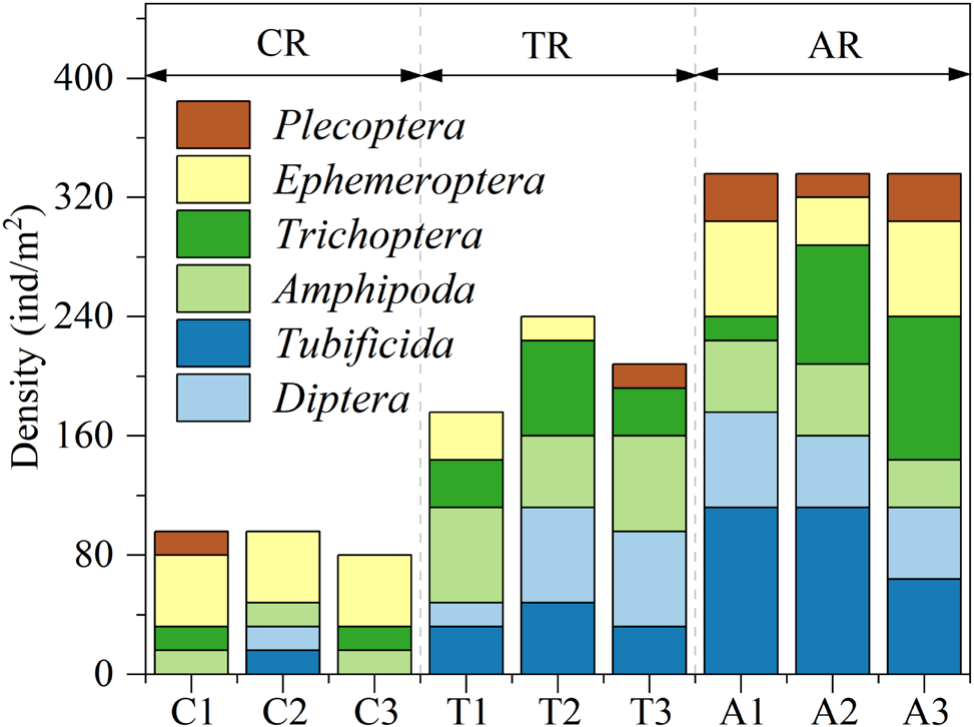
Benthic macroinvertebrate density distribution across three river reach types in the upper Yangtze River. Sampling sites are classified into three reach types: C1–C3 (Canyon reach, CR), T1–T3 (Reef–Tuo reach, TR), and A1–A3 (Anabranching reach, AR).

At the order level, community composition varied significantly across the three reaches (Table 1 and Figure 2). In the Canyon reach, the community was primarily dominated by Ephemeroptera and Plecoptera, particularly the families Baetidae and Hydropsychidae. In contrast, the Reef–Tuo reach exhibited a more balanced assemblage, with relatively even contributions from Diptera, Trichoptera, and Amphipoda. Dominant taxa included the families Rhyacophilidae, Brachycentridae, and *Procladius* sp., as well as *Gammarus sinensis*. In the Anabranching reach, the community was primarily dominated by Trichoptera and Oligochaeta, with the families Rhyacophilidae, *Limnodrilus hoffmeisteri* , and Naididae being particularly abundant. At the same time, Amphipoda and Ephemeroptera were also prominent, with *Gammarus sinensis* and Baetidae occurring in high numbers.

### 3.2 Benthic Macroinvertebrate Diversity Across Habitat Types at Different River Segment Scales

**FIGURE 3:**
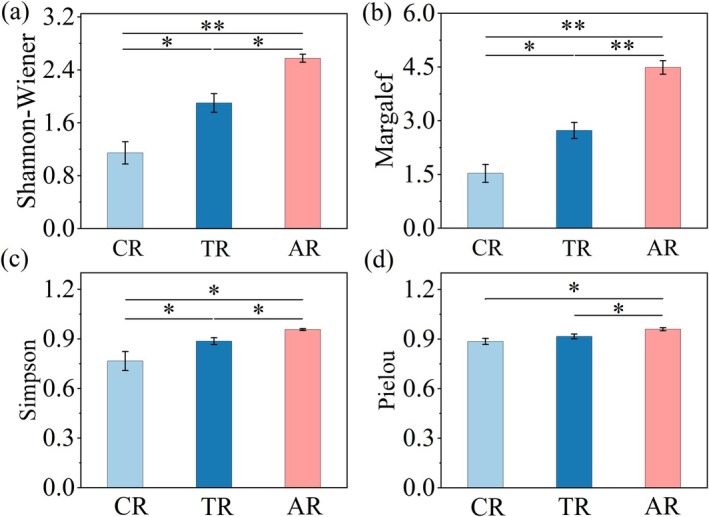
Diversity indices of benthic macroinvertebrate communities across three river reach types in the upper Yangtze River. (a) Shannon–Wiener, (b) Margalef, (c) Simpson, and (d) Pielou indices are shown. CR = Canyon reach; TR = Reef–Tuo reach; AR = Anabranching reach. * indicate significant differences between reaches (*p < 0.05, **p < 0.01).

The community diversity indices for the three habitat types are shown in Figure 3. The Anabranching reach demonstrates the highest values, with a Margalef richness index (*D*_M_ = 4.58), a Shannon–Wiener diversity index (*H*′ = 2.82), a Simpson diversity index (*D* = 0.94), and a Pielou evenness index (*J* = 0.95). The Reef–Tuo reach follows with (*D*_M_ = 3.83, *H*′ = 2.21, *D* = 0.89, *J* = 0.86). In contrast, the Canyon reach shows the lowest values across all diversity indices: *D*_M_ = 1.76, *H*′ = 1.39, *D* = 0.71, and *J* = 0.79.

The results of one‐way ANOVA showed significant differences in the Margalef, Shannon–Wiener, and Simpson indices among the three habitat types (*p* < 0.05). However, no significant difference was found in the Pielou index between the Reef–Tuo and Canyon reaches (*p* > 0.05). In addition, Tukey's post hoc test revealed significant differences in the Shannon–Wiener and Margalef indices between the Anabranching and Canyon reaches (*p* < 0.01), and in the Margalef index between the Reef–Tuo and Anabranching reaches (*p* < 0.01).

### 3.3 Differences in Benthic Community Structure Among Habitat Types

**FIGURE 4:**
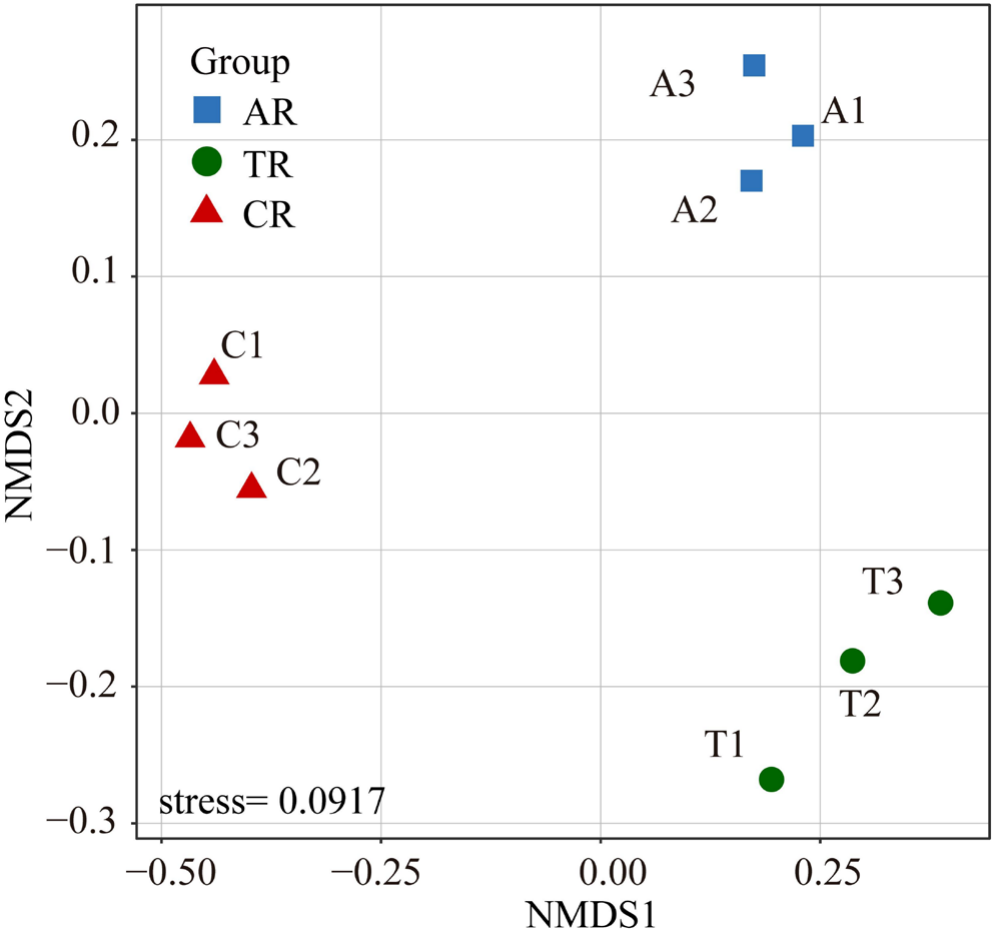
NMDS ordination illustrating differences in benthic macroinvertebrate community structure among three typical riverine habitat types: Canyon reach (CR), Anabranching reach (AR), and Reef–Tuo reach (TR). Each point represents a sampling site; shorter distances between points indicate higher similarity in community composition.

NMDS analysis (Figure 4) revealed clear spatial separation of the benthic invertebrate communities among the three habitat types—Canyon reach (CR), Anabranching reach (AR), and Reef–Tuo reach (TR). Within each habitat type, the three replicate sampling sites (e.g., C1–C3) are closely clustered, indicating that sites within the same habitat type have highly similar community compositions. This spatial clustering aligns with expectations, suggesting that river reaches with similar geomorphology and hydrodynamics harbor similar macroinvertebrate communities.

To further assess the significance of the differences in benthic community structure among the river reach types, we conducted ANOSIM and PERMANOVA analyses. ANOSIM results show significant differences in community structure between the river reach types (Canyon reach, Anabranching reach, and Reef–Tuo reach) (*R* = 0.975, *p* = 0.007), with much greater between‐group than within‐group variation. Permutation tests confirmed that the observed *R* value was far higher than the 99% quantile (*R* = 0.790), further validating the significant community structure differentiation. The within‐group distance rank ranges were relatively small (e.g., 1.0–5.0 for Canyon reach), whereas the between‐group distances were significantly higher (9.0–36.0), consistent with the NMDS visualization. Further PERMANOVA analysis indicated that habitat type had a significant effect on community structure (*F* = 10.047, *p* = 0.004), explaining approximately 77% of the variation in community differences (*R* ^2^ = 0.770), confirming that the community structure differs significantly between different habitat types.

### 3.4 Relationship Between Benthic Community Structure and Environmental Factors

One‐way ANOVA and Kruskal‐Wallis H tests revealed significant differences in several physico‐chemical parameters among river reach types (*p* < 0.05), including V, Turbidity, TNs, TPs, and TOC. The Canyon reach showed significantly higher flow velocities, while the Reef–Tuo and Anabranching reaches exhibited higher sediment nutrient and organic matter levels (Table 2).

**TABLE 2:** Environmental characteristics and significant differences of three typical river sections.

Categories	Variables	Canyon reach	Reef–Tuo reach	Anabranching reach	*p*	
					ANOVA	Kruskal‐Wallis test
Physical parameters	T(‐°C)	19.60 ± 0.76	19.59 ± 1.13	19.60 ± 0.98	0.472	
	Turbidity (NTU)	21.64 ± 5.37	15.21 ± 0.57	17.18 ± 2.70	/	**0.008** *
	V(m/s)	0.98 ± 0.43	0.29 ± 0.19	0.55 ± 0.27	**0.001** *	
	TSS (mg/L)	7.60 ± 1.50	7.00 ± 1.63	6.25 ± 0.43	0.435	
Chemical parameters	DO(mg/L)	7.61 ± 0.32	7.62 ± 0.84	7.63 ± 0.47	0.178	
	CHL(*μ*g/L)	0.35 ± 0.03	0.36 ± 0.01	0.36 ± 0.02	0.129	
	pH	8.19 ± 0.27	8.19 ± 0.23	8.19 ± 0.30	0.902	
	SpCond (μS/cm)	399.17 ± 4.97	400.32 ± 5.58	400.27 ± 3.69	0.531	
	TN (mg/L)	1.98 ± 0.24	2.12 ± 0.29	2.03 ± 0.17	0.782	
	TP (mg/L)	0.07 ± 0.01	0.09 ± 0.01	0.08 ± 0.02	0.287	
	COD_Mn_ (mg/L)	2.70 ± 0.18	2.87 ± 0.12	2.70 ± 0.39	/	0.720
	NH_4_^+^‐N (mg/L)	0.28 ± 0.03	0.29 ± 0.02	0.29 ± 0.05	0.930	
Sediment parameters	TNs (mg/kg)	510.04 ± 18.4	799.27 ± 23.06	677.07 ± 60.3	**0.002** *	
	TPs (mg/kg)	535.52 ± 29.2	794.50 ± 74.38	863.92 ± 25.0	/	**0.003** *
	TOC (g/kg)	7.32 ± 1.61	16.45 ± 0.55	18.13 ± 2.27	**0.002** *	

* *p* < 0.05, significant difference.

**FIGURE 5:**
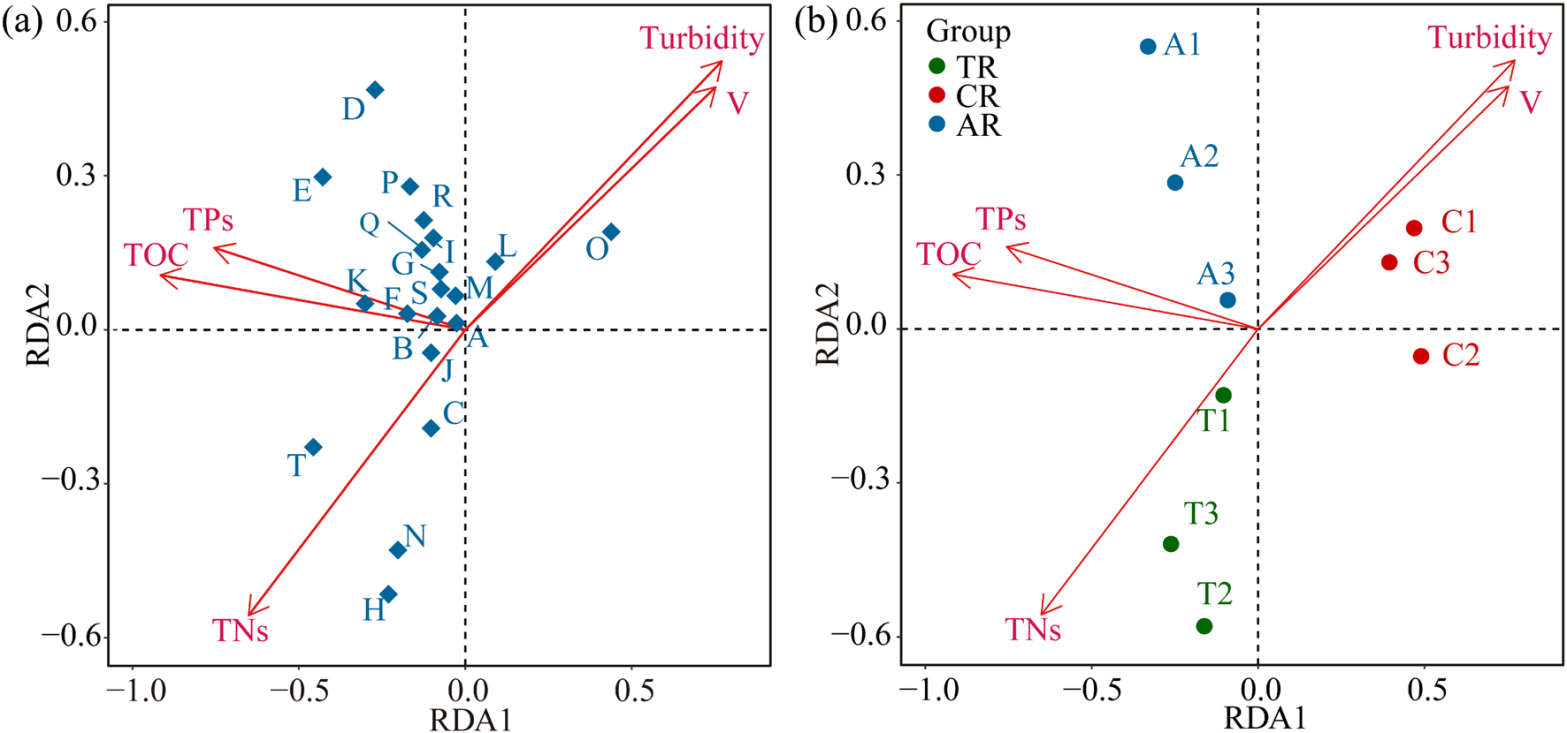
Redundancy analysis (RDA) ordination plot illustrating the relationship between environmental variables and benthic macroinvertebrate community structure. (a) The direction and length of the arrows represent the influence of each environmental variable (TOC, TNs, TPs, etc.) on community composition. (b) Environmental variables and sampling sites are shown with distinct groupings: CR (Canyon reach, red), TR (Reef–Tuo reach, green), and AR (Anabranching reach, blue). The direction and strength of the environmental variables' influence are indicated by the arrows.

RDA was used to explore the relationship between environmental factors, which varied significantly across habitat types, and benthic community structure. The first two ordination axes explained 82.6% of the variation in community structure, with the first axis accounting for the largest proportion (Figure 5). V (*r* = 0.93) and Turbidity (*r* = 0.78) were strongly correlated with the distribution of samples from the Canyon reach. TOC (*r* = −0.89) and TPs (*r* = −0.78) were closely associated with the Anabranching reach samples, while TNs (*r* = −0.74) was most closely related to the community distribution in the Reef–Tuo reach. These findings highlight the combined effects of physical hydrodynamic conditions and sediment nutrient status on community differentiation.

### 3.5 Identification of Major Drivers and Response Pathways

In this study, we divided the collected species into two categories: rheophilic taxa and sediment‐preferring taxa. Five environmental factors with significant differences were selected: Velocity, Turbidity, and Sediment condition (including TOC, TNs, and TPs) as exogenous variables. Partial Least Squares Path Modeling (PLS‐PM) was used to analyze the direct and indirect pathways of these environmental factors on the two benthic organism groups.

**FIGURE 6:**
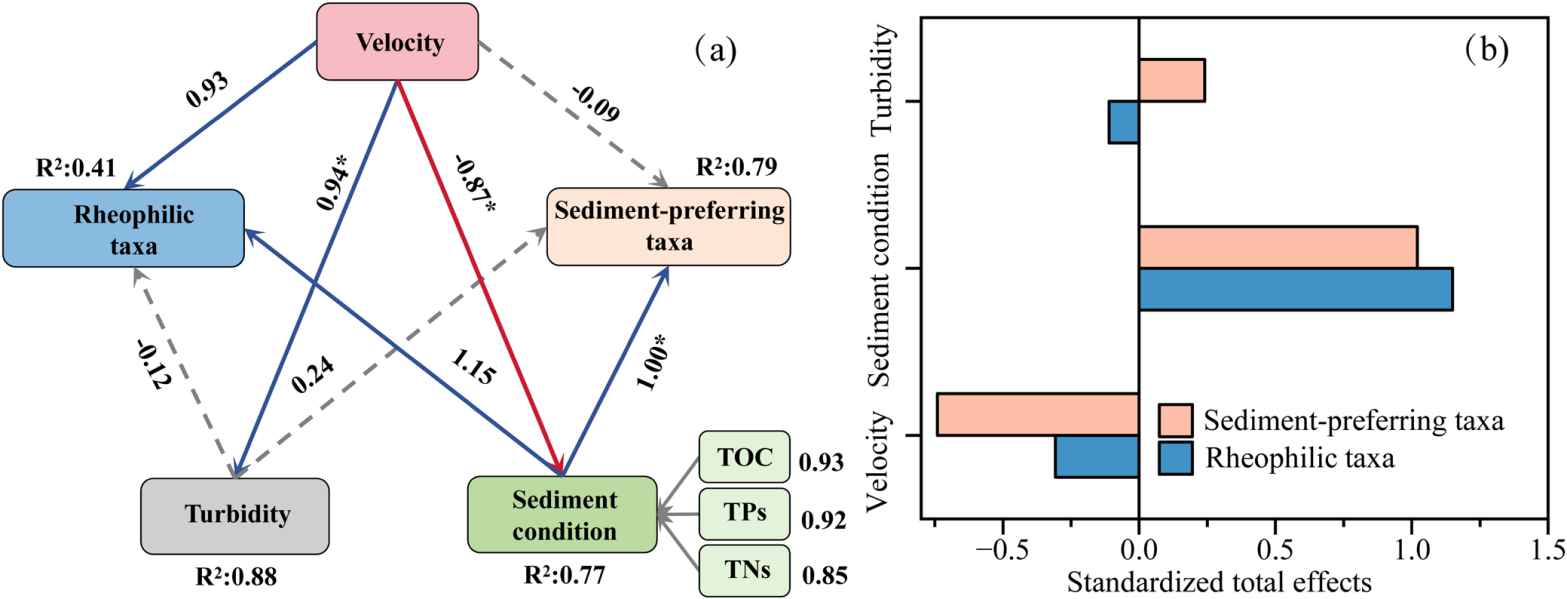
Partial least squares path modeling (PLS‐PM) showing the effects of environmental factors on functional benthic groups. (a) Structural model illustrating the direct and indirect effects of flow velocity, turbidity, and sediment conditions (TOC, TNs, TPs) on rheophilic taxa and sediment‐preferring taxa. Solid arrows indicate significant paths (*p < 0.05); blue arrows represent positive effects, red arrows represent negative effects, and dashed arrows indicate non‐significant paths. (b) Standardized total effects of the three environmental modules on the two functional groups.

The PLS‐PM results (Figure 6a) indicated that velocity exhibited a relatively high overall influence among the environmental factors and showed a strong positive relationship with rheophilic taxa (path coefficient = 0.93). In contrast, the direct effect of velocity on sediment‐preferring taxa was relatively weak (path coefficient = −0.09), but it indirectly enhanced ecological support for these taxa by significantly improving sediment conditions (path coefficient = 1.0). Further standardized total effect analysis (Figure 6b) revealed that although the direct effect of velocity on rheophilic taxa was substantial, its total effect remained relatively balanced, likely due to partial mediation through sediment or other environmental factors. In comparison, the influence of velocity on sediment‐preferring taxa was mainly realized through the indirect pathway of sediment condition regulation.

Overall, velocity influenced the benthic community structure through dual pathways of “direct filtering” and “indirect mediation”, simultaneously promoting the aggregation of rheophilic taxa in high‐velocity environments and facilitating the persistence and dispersal of sediment‐preferring taxa by improving habitat substrate conditions.

### 3.6 Hydrodynamic Characteristics of River Sections With Different Habitat Types

**FIGURE 7:**
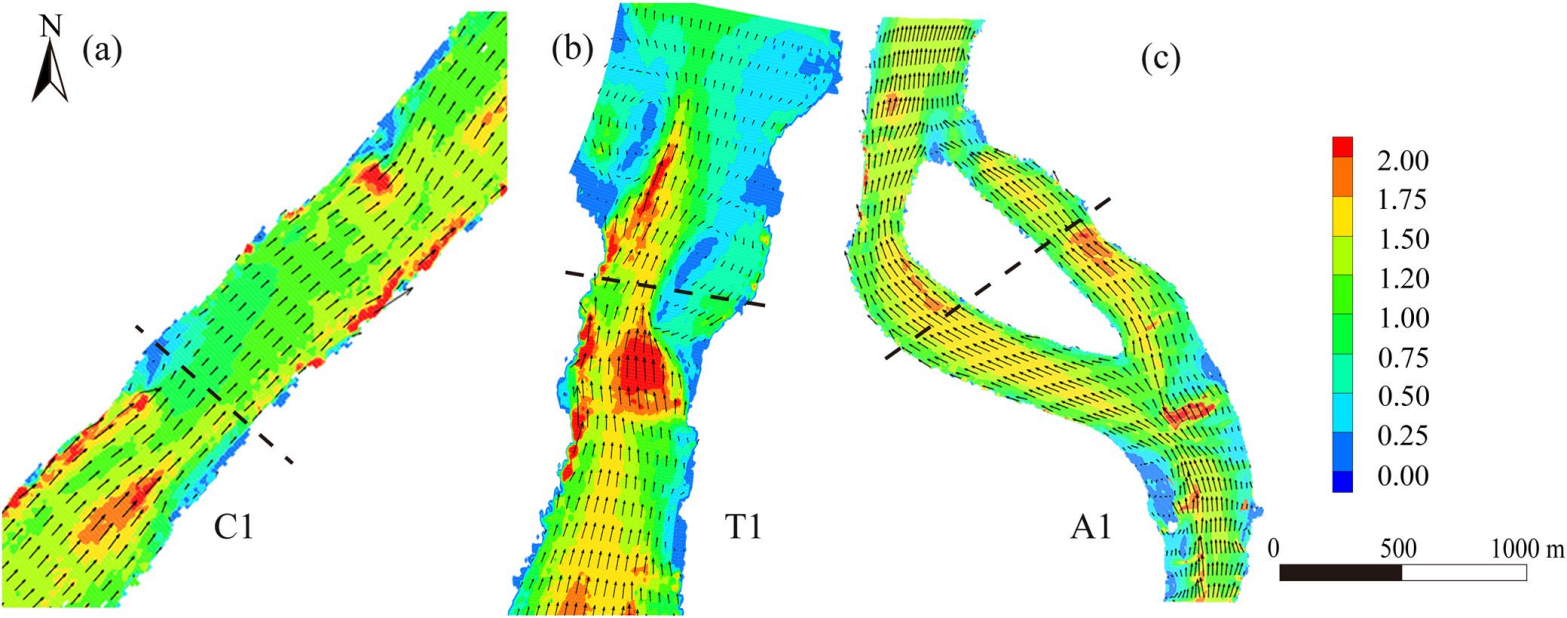
Hydrodynamic simulation results for three typical river reaches in the study area. (a–c) Two‐dimensional flow velocity distributions under steady‐state conditions for typical river reaches, representing the Canyon reach (C1), Reef–Tuo reach (T1), and Anabranching reach (A1). The color gradient indicates flow velocity (m/s), and the arrows represent flow direction and relative velocity magnitude.

The hydrodynamic model simulations revealed significant differences in flow velocity distribution across the three river reach types (Figure 7). In the Canyon reach, flow is concentrated in the deep main channel, with peak velocities reaching 2.5 m/s. Along the bedrock‐bound banks, velocities remain between 1.2 and 1.8 m/s, resulting in a high‐velocity distribution throughout the reach. In the Anabranching reach, velocities decrease notably, ranging from 0.8 to 1.2 m/s in the main channel and from 0.3 to 0.6 m/s at the confluence of the bars and channels, forming a main‐slow flow transition zone. In the Reef–Tuo reach, the main channel has velocities between 1.5 and 2.0 m/s, while most of the Tuo area maintains velocities of 0.3 to 0.8 m/s. A clear velocity gradient exists between the main channel and slow‐flow areas, with local recirculation zones, resulting in a high‐low velocity flow pattern.

Simulations of flow velocity distribution were conducted for the Reef–Tuo reach under three typical discharge conditions: 9920, 14,600, and 30,400 m^3^/s. In the Reef–Tuo zone, flow velocities ranged from 0.3 to 0.8 m/s, with little variation in the position of the main flow and recirculation boundaries, indicating a stable flow structure. The main channel showed a notable response to discharge changes, with peak velocities exceeding 2.0 m/s under high‐flow conditions. This reach displays a composite flow pattern, characterized by stable Tuo areas and sensitive main channels.

## 4 Discussion

**FIGURE 8:**
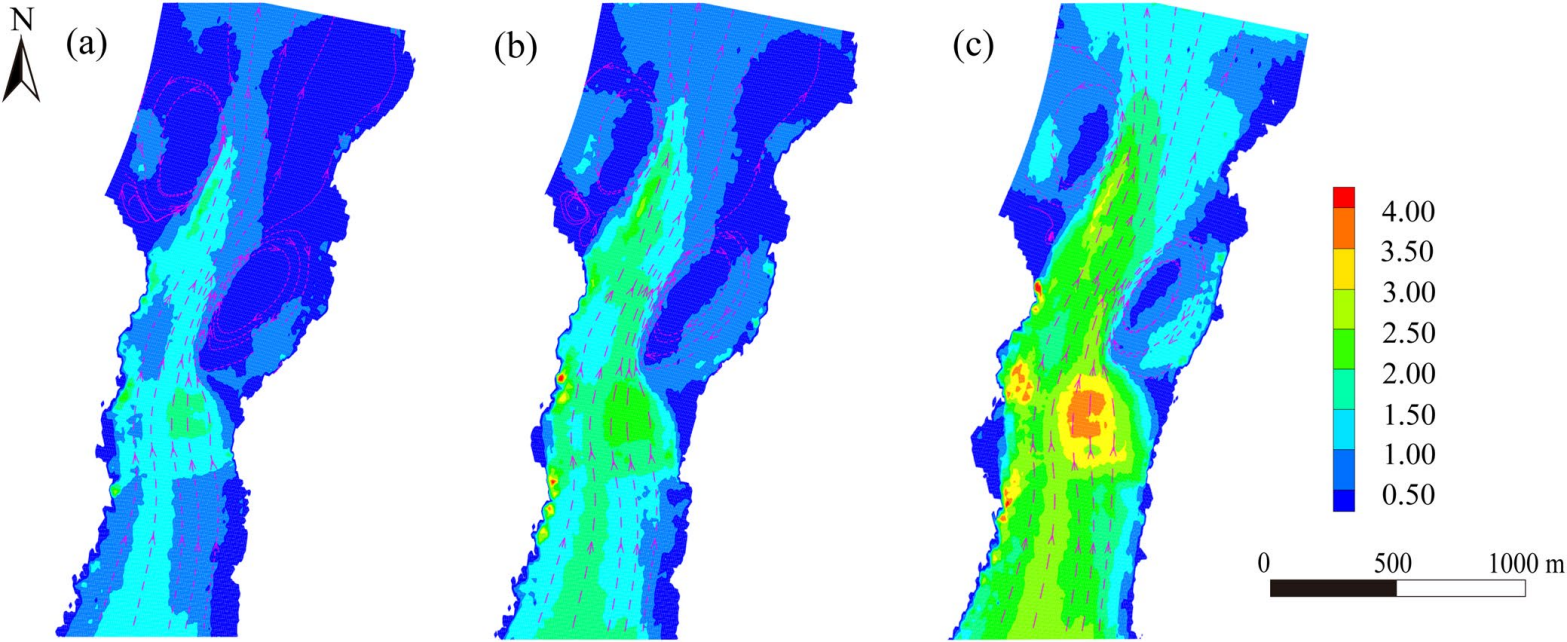
Hydrodynamic simulation results under three different discharge conditions in the study area. (a–c) Simulated flow velocity distributions for the Reef–Tuo reach (T1) under three different discharge conditions: 9920 m^3^/s, 14600 m^3^/s, and 30,400 m^3^/s.

This study investigates how habitat heterogeneity at the river reach scale shapes the assembly of benthic macroinvertebrate communities. Unlike conventional ecological research that emphasizes microhabitats (e.g., riffles, pools, substrate composition) or broad watershed‐scale patterns (Dos Reis Oliveira et al. 2020; Hitchman et al. 2018), our work focuses on meso‐scale hydrodynamic variation—specifically, spatial heterogeneity within river reaches. We propose a dual‐pathway ecological assembly framework in which reach‐scale morphology shapes flow velocity distribution and triggers two distinct but interacting ecological processes: direct ecological filtering and indirect sediment–nutrient coupling, which jointly determine benthic community structure (Figure 8). Grounded in a synthesized understanding of environmental filtering and resource‐mediated regulation, this study emphasizes that these processes can function synergistically under a unified hydrodynamic regime—revealing a spatial coupling among physical habitat structure, ecological processes, and community‐level functioning. In contrast to previous models that treated these pathways independently (Cheng et al. 2019; Pinna et al. 2024), we propose a spatially integrative framework rooted in reach‐scale habitat heterogeneity.

Redundancy analysis (RDA; Figure 5) identified flow velocity, turbidity, and benthic sediment nutrients (total organic carbon, TOC; total nitrogen, TNs; total phosphorus, TPs) as the principal environmental variables structuring benthic communities, with flow velocity showing the highest explanatory power (*r* = −0.9252), underscoring its dominant role in ecological filtering. The structural equation model (PLS‐PM) (Figure 6) further corroborated this pattern: flow velocity exerted a significant positive effect on rheophilic taxa (path coefficient = 0.93). In Canyon reaches, velocities were strongly concentrated, peaking at 2.5 m/s in the main channel and remaining at 1.2–1.8 m/s along the banks, forming a high‐velocity‐dominated hydraulic regime. Under such conditions, most benthic taxa are unable to attach or reproduce, and only species with strong attachment and resistance to flow—such as larvae of the family Baetidae—can persist. Baetid larvae possess flattened bodies and specialized attachment structures that enable them to thrive in fast currents, often becoming local dominants (Luo et al. 2018). This filtering mechanism acts not only on species‐level survival strategies but also drives functional trait differentiation at the community level, highlighting flow velocity's central role as an ecological filter in community assembly.

**FIGURE 9:**
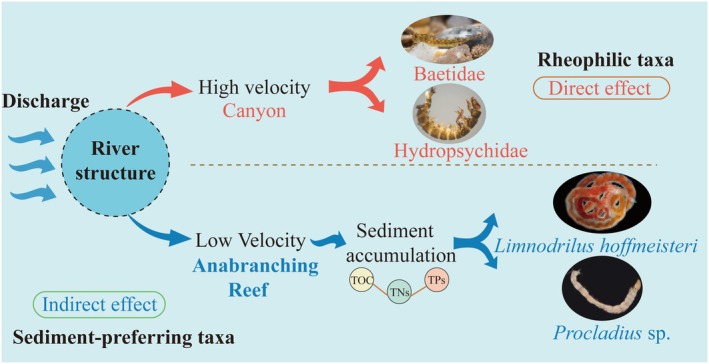
Schematic representation of the direct and indirect effects of river structure on benthic macroinvertebrate assemblages under varying hydrological conditions.

Beyond directly selecting rheophilic taxa, the structural equation model also revealed an indirect pathway by which flow velocity influences sediment‐preferring taxa (Figure 6): velocity modulates benthic nutrient conditions, which in turn significantly affect the composition of sediment‐preferring taxa. Benthic nutrient factors exerted the strongest direct effect on sediment‐preferring taxa (path coefficient = 1.0, *p* < 0.1). In Anabranching and Reef–Tuo reaches, reduced main‐channel velocity leads to locally elevated turbidity and enhanced fine‐sediment deposition. However, in contrast to findings from small streams where deposition suppresses community development (Pilkerton et al. 2025), we found that lateral backflow, periodic disturbances, and localized turbulence in large‐river low‐velocity zones help maintain substrate permeability and benthic oxygen supply (Blöcher et al. 2020), while increasing TOC, TN, and TP availability. These conditions provide stable resources for sediment‐preferring taxa such as *Limnodrilus hoffmeisteri* and *Procladius* sp., thereby enhancing their diversity and density (Harrison et al. 2023; Qi et al. 2019). The results indicate that flow velocity plays a central role in benthic community assembly through a dual‐pathway mechanism: it acts as a direct ecological filter selecting rheophilic taxa, and it indirectly promotes the development of sediment‐preferring taxa by influencing sediment deposition and nutrient supply via hydrodynamic disturbances (Figure 9). Although turbidity and sediment variables also show high explanatory power in the RDA, their strong collinearity with velocity may cause statistical masking, obscuring their independent ecological effects. Accordingly, the structural model provides a clearer identification of causal paths, emphasizing that velocity both drives functional trait selection and regulates nutrient coupling under specific habitat conditions, forming an integrated “velocity–microhabitat–community response” framework (Wang, Bao, et al. 2024; Wang, Guo, et al. 2024). This dual‐pathway mechanism deepens understanding of structure–function relationships in large river ecosystems and offers theoretical support for habitat restoration and ecological management.

During the study period, flow fluctuations were minimal, and local hydrodynamic changes were primarily controlled by riverbed topography. Riverbed cross‐sectional morphology, as a key physical variable, regulates local flow velocity patterns, which in turn affect sediment deposition and nutrient conditions, ultimately driving spatial heterogeneity in community structure (Cheng et al. 2019; Wang et al. 2025) Two‐dimensional flow field simulations (Figure 7) further reveal this process: Canyon reach shows concentrated flow velocities, Anabranching reach exhibits dispersed flow velocities with high heterogeneity, while Reef–Tuo reach shows low‐flow zones and backwater areas. This spatial heterogeneity effectively reduces sediment loss under high‐flow conditions, ensuring the stability of sedimentary environments. The complexity of the riverbed cross‐section enhances local flow velocity gradients by altering flow conditions, which further strengthens the dual‐pathway ecological effect and amplifies the influence of hydrological conditions on benthic communities.

To compensate for the limitation of single hydrological conditions during the field sampling period, this study selected the Reef–Tuo Reach (T1) and conducted flow field simulations under three representative discharge conditions: 9920, 14,600, and 30,400 m^3^/s (Figure 8). The simulation results indicated that even under the high‐flow scenario of 30,400 m^3^/s, low‐velocity zones within the reach remained stable. This hydrodynamic feature effectively attenuated the impact of peak flows on habitat structures and sedimentary conditions, providing relatively stable refugia for benthic organisms. Such stable low‐velocity environments not only offered continuous nutrient supply and shelter for sediment‐preferring taxa but also supported the survival and recovery of rheophilic taxa such as Baetidae and Hydropsychidae during flood peaks (Su et al. 2019). These taxa were also widely observed in our study, further confirming the crucial role of low‐velocity habitats in sustaining benthic biodiversity. More importantly, the persistence of flow velocity heterogeneity and backwater structures under all flow conditions provides a robust physical foundation for the “dual‐pathway mechanism”. This indicates that the mechanism is not a flow‐specific or transient phenomenon but rather a stable ecological response driven by the intrinsic coupling between channel morphology and local hydrodynamics. This conclusion aligns closely with the “refuge effect” in ecological resilience theory, which suggests that spatial heterogeneity preserves low‐disturbance ecological niches, thereby supporting long‐term population persistence and facilitating rapid recovery following disturbance (Mao et al. 2023; Sanches et al. 2023). Meanwhile, the presence of flow gradients also promotes the coupling of sediment and nutrient processes, sustaining benthic community growth and reproduction under high‐flow conditions. This further reinforces the structural stability of the ecosystem (Yu et al. 2025) and highlights the broad applicability of the dual‐pathway mechanism across fluctuating hydrological regimes.

## 5 Conclusions and Perspectives

### 5.1 Conclusions

This study elucidates key ecological processes and frameworks underpinning community assembly and diversity maintenance of benthic macroinvertebrates in a large mountainous river in the upper Yangtze River. Main conclusions include: (1) reach‐scale habitat heterogeneity significantly influences community composition and ecological response patterns; (2) flow velocity drives community structure via a dual‐pathway mechanism involving direct ecological filtering and indirect sediment‐nutrient interactions; and (3) habitat heterogeneity at the reach scale enhances ecosystem resilience under extreme environmental conditions. These findings advance mesoscale ecological theory by clarifying the pivotal ecological role of reach‐scale habitats in linking microhabitat and catchment‐scale processes, providing important theoretical foundations for ecological conservation and habitat management in the upper Yangtze River.

### 5.2 Limitations and Future Directions

Several limitations remain: (1) absence of long‐term ecological monitoring data to reveal community resilience dynamics under extreme disturbance events; and (2) limited integration of functional traits and ecosystem processes, restricting deeper insights into ecological functionality.

Future research should focus on: (1) implementing long‐term ecological monitoring to understand community resilience mechanisms under extreme disturbances; (2) combining taxonomic and functional trait analyses to investigate the impacts of reach‐scale habitat structures on ecosystem function; and (3) developing an integrated ecological framework that connects microhabitat, reach‐scale, and catchment‐scale heterogeneity to comprehensively explore multi‐scale mechanisms of community assembly.

## Author Contributions

**Yang Wei:** conceptualization (lead), data curation (equal), formal analysis (supporting), funding acquisition (lead), investigation (equal), writing – original draft (lead), writing – review and editing (lead). **Wang Si‐Yuan:** conceptualization (equal), data curation (lead), formal analysis (lead), investigation (equal), methodology (equal), software (equal), writing – original draft (lead), writing – review and editing (supporting). **Zhang Xian‐Bing:** conceptualization (equal), formal analysis (supporting), funding acquisition (equal), investigation (supporting), project administration (supporting), supervision (equal), writing – review and editing (supporting). **Zhang Yang‐Chun:** methodology (equal), writing – original draft (supporting), writing – review and editing (supporting). **Chen Zi‐Wei:** project administration (equal), resources (equal), supervision (equal), writing – review and editing (equal). **Yang Zhong‐Chao:** formal analysis (equal), software (equal), writing – review and editing (supporting). **Yang Sheng‐Fa:** funding acquisition (equal), resources (equal), writing – review and editing (supporting). **Chen Qi‐Liang:** writing – review and editing (equal). **Xie Jia‐Hui:** investigation (equal), writing – review and editing (supporting). **Tan Li‐Wei:** investigation (equal), writing – review and editing (supporting).

## Conflicts of Interest

The authors declare no conflicts of interest.

## Data Availability

Data are stored on Figshare (https://doi.org/10.6084/m9.figshare.30247522.v1).
